# Prognostic impact of spread through air spaces in lung adenocarcinoma

**DOI:** 10.1093/icvts/ivab289

**Published:** 2021-10-18

**Authors:** Sara Mantovani, Angelina Pernazza, Massimiliano Bassi, Davide Amore, Jacopo Vannucci, Camilla Poggi, Daniele Diso, Giulia d’Amati, Carlo Della Rocca, Erino Angelo Rendina, Federico Venuta, Marco Anile

**Affiliations:** Department of Thoracic Surgery, University of Rome Sapienza, Rome, Italy; Department of Medical-Surgical Sciences and Biotechnologies, University of Rome Sapienza, Rome, Italy; Department of Thoracic Surgery, University of Rome Sapienza, Rome, Italy; Department of Thoracic Surgery, University of Rome Sapienza, Rome, Italy; Department of Thoracic Surgery, University of Rome Sapienza, Rome, Italy; Department of Thoracic Surgery, University of Rome Sapienza, Rome, Italy; Department of Thoracic Surgery, University of Rome Sapienza, Rome, Italy; Department of Radiological, Oncological and Pathological Sciences, University of Rome Sapienza, Rome, Italy; Department of Radiological, Oncological and Pathological Sciences, University of Rome Sapienza, Rome, Italy; Department of Thoracic Surgery, University of Rome Sapienza, Rome, Italy; Department of Thoracic Surgery, University of Rome Sapienza, Rome, Italy; Department of Thoracic Surgery, University of Rome Sapienza, Rome, Italy

**Keywords:** Adenocarcinoma, STAS, Pulmonary resections, Lymph node involvement

## Abstract

**OBJECTIVE:**

Spread through air spaces (STAS) is a pattern of invasion present in some adenocarcinomas (ADC). The goal of this study was to assess the impact of STAS in patients treated with different types of surgical resections and on the clinical outcome in patients with ADC of different diameters and with different degrees of nodal involvement.

**METHODS:**

A total of 109 patients were reviewed. Complete surgical resection with systematic nodal dissection was achieved in all patients. The median follow-up was 65 months (3–90 months).

**RESULTS:**

STAS was observed in 70 cases (64.2%); 13 patients (18.5%) had lymph node involvement (N1 and N2). Overall survival and progression-free survival were higher in patients without STAS (*P* = 0.042; *P* = 0.027). The presence of STAS in tumours ≤2 cm was a predictor of worse progression-free survival following sublobar resection compared to major resections (*P* = 0.011). Sublobar resection of N0 STAS-positive tumours was associated with worse long-term survival compared to a major resection (*P* = 0.04). Statistical analyses showed that age >70 years and recurrence were independent variables for survival; smoking pack-years >20, sublobar resection and nodal involvement were independent variables for recurrence; and smoking pack-years >20 were independent variables for a history of cancer and pleural invasion for local recurrence.

**CONCLUSIONS:**

STAS seems to play a role in long-term survival, particularly for patients with N0 and tumours smaller than 2 cm. Further studies are necessary to validate this hypothesis.

**Figure:**
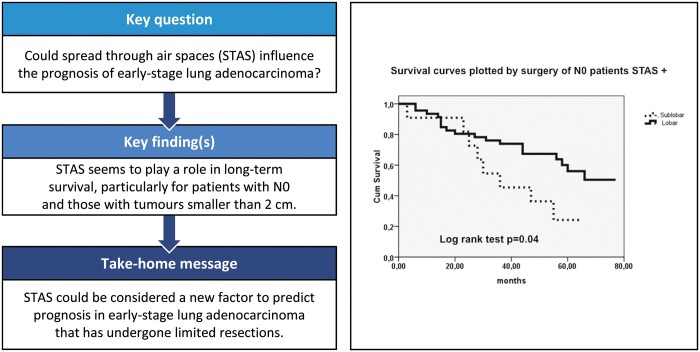

## INTRODUCTION

Spread through air spaces (STAS) of adenocarcinoma (ADC) is a peculiar pattern of local invasion acknowledged by the last World Health Organization classification [1]. According to the original definition, it comprises tumour cells (forming either micropapillary structures or solid nests or appearing as isolated cells) spreading within air spaces beyond the tumour edge without direct connection with the primary tumour [2].

Although this concept has been challenged by some authors [3], STAS should be considered a negative prognostic factor in stage I ADC; it is able to predict recurrence in case of both sublobar resection and lobectomy [2, 4, 5]. However, some points still remain controversial. In fact, the exact mechanism underlying STAS and the prognostic impact at more advanced stages are still to be defined. In addition, although it has been recently reported that STAS is a predictor of occult lymph node metastases in clinical stage I ADC [6], the relationship with lymph node involvement has not been clarified. Finally, the gold standard treatment for STAS positive ADC is still to be defined [7].

The goal of this retrospective study was to assess the prognostic impact of STAS in patients undergoing different types of surgical resections and to correlate it with the diameter of the tumour and the extent of nodal involvement.

## MATERIALS AND METHODS

The database included 124 patients with lung ADC undergoing pulmonary resection. Fifteen patients were lost to follow-up; the remaining patients were assessed by a clinical visit or a telephone interview. Inclusion criteria were age >18 years and pulmonary resection for ADC. The study period included cases treated and retrospectively reviewed by pathologists from January 2013 to September 2015. The mean age was 68.9 ± 10.1 years; the male/female ratio was 1.4/1 (63/46); 38 (35%) were smokers, 48 (44%) were former smokers and 23 (21%) never smoked. Fifty-seven patients (52.3%) had a history of cancer, and 30 of them (53%) had previously undergone chemotherapy or chemoradiotherapy; 6 patients (5.5%) received induction therapy for preoperative mediastinal lymph node involvement. All patients underwent the standard preoperative work-up, which included routine blood tests, radiological evaluation (total body computed tomography and positron emission tomography), blood gas analysis, pulmonary function tests, cardiologic assessment with an electrocardiogram and echocardiography; further examinations such as a cardiac stress test or an exercise test to assess VO_2_max were performed when required.

### Ethical statement

All patients signed an informed consent form before the operation. This retrospective study was approved by our institutional ethics committee (No 02/2019).

### Surgical treatment

Eight-three patients (76.1%) had major pulmonary resections including 75 lobectomies (90.4%), 5 bilobectomies (6%) and 3 pneumonectomies (3.6%). In 26 cases (23.9%), we performed sublobar resections (23 wedge resections and 3 segmentectomies) due exclusively to poor preoperative cardiopulmonary function. In particular, no histological differences were detected between the 2 surgical groups. Complete surgical resection was achieved in all patients; a free margin of at least 1.5 cm was recorded for sublobar resections. According to the European Society of Thoracic Surgeons guidelines [8], a systematic nodal dissection was performed routinely, with a median of 8 lymph nodes removed (range 6–13).

### Histological evaluation

**Figure 1:**
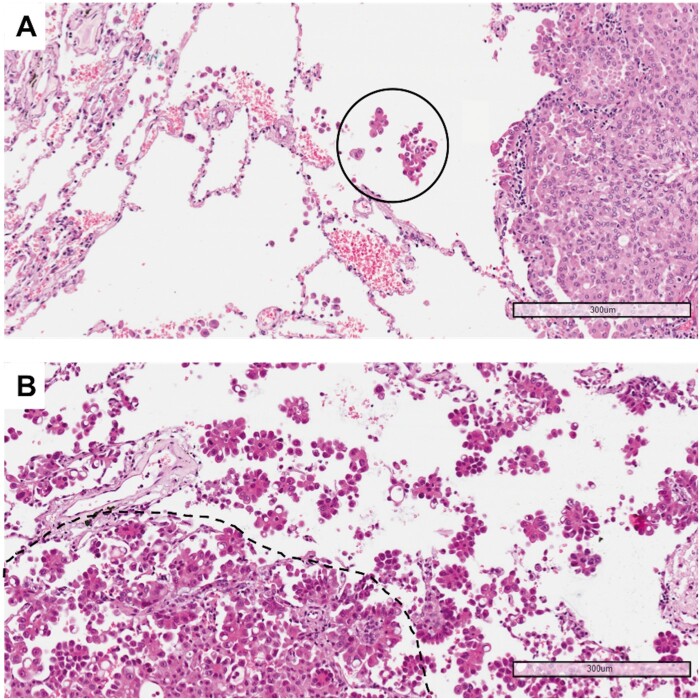
(**A**) Example of a tumour with low spread through air spaces: only 1 solid nest of neoplastic cells (circle) is detectable in the alveolar spaces beyond the edge of the tumour (haematoxylin and eosin, 10×). (**B**) Example of a tumour with high spread through air spaces: multiple micropapillary clusters are present beyond the edge of the tumour (dashed line) (haematoxylin and eosin, 10×).

The requirement to reliably evaluate STAS in the surgical specimen was the presence of a circumferential rim of normal lung (at least 1 cm) surrounding the edge of the tumour. Accordingly, we had previously selected from our retrospective cases those in which the tumour periphery adjacent to normal lung tissue was present in at least 1 histological slide. From 2 to 7 haematoxylin and eosin slides were available from each sample and were reviewed by 2 expert pathologists (Gd’A and AP). Cases were divided into 2 groups: STAS positive and STAS negative. In STAS positive tumours, the morphological pattern was detailed as follows: micropapillary clusters, solid nests or single cells. Finally, to assess whether the extension of STAS could be a prognostic factor, the cases were categorized as low STAS (involving up to 2/3 of the tumour circumference) and high STAS (involving the entire tumour circumference) (Fig. 1).

### Statistical analysis

The independent variables analysed for survival and recurrence were age (cut-off 70 years), gender, history of smoking, previous history of malignancy, comorbidities, radiological features of ground-glass opacity, diameter of the tumour (cut-off 20 mm), lymph node involvement, staging, grade of differentiation of cancer, presence of STAS, extension of STAS (low versus high), lymphatic invasion, pleural invasion, histological patterns (acinar, lepidic, papillary, solid, micropapillary, cribriform, mucinous), induction therapy, adjuvant therapy and type of surgery (sublobar resection versus other). Continuous variables were shown as mean ± standard deviation whereas categorical variables were presented as number and percentages; the *Student's t-test* and the χ^2^ test were used according to the variables. Cox regression analysis was performed to assess the impact of the different variables on survival and on the incidence of local and overall recurrence (considered local plus distant). The overall and the progression-free survival curves were determined using a Kaplan–Meier analysis. The log-rank test was used to compare survivals. A *P*-value <0.05 was considered statistically significant. The statistical analysis was performed using SSPS 17.0 software (IBM-SPSS Statistics, Armonk, NY, USA).

## RESULTS

The median follow-up was 65 months (3–90 months). Demographic data are reported in Table 1. No perioperative deaths were observed; the mean time from the operation to hospital discharge was 7.8 ± 4.6 days. Twenty-two patients (20.2%) showed postoperative complications, mainly persistent air leaks (9–40.9%) and atelectasis (4–18.1%). According to the 8th staging system, 72 patients (66.1%) were stage I; 17 (15.6%) were stage II; and 20 (18.3%) were stage IIIA. Twenty-three patients (21.1%) received adjuvant chemotherapy; 11 of them (47.8%) also received radiotherapy. We observed 37 recurrences (34%); 14 of them (37.3%) were considered local.

**Table 1:** Characteristics of the study cohort

Variables	Number of patients	Percentage
Age, median (range)	68.9 (39–86)	
<70	59	54.1
≥70	50	45.9
Sex		
Male/female	63/46	57.8/42.2
Smokers		
No	23	21.1
Yes-Ex	86	78.9
Surgery		
Major resection	83	76.1
Sublobar resection	26	23.9
Tumour size		
<20 mm	48	56
≥20 mm	61	44
N stage		
N0	92	84.4
N1–N2	17	15.6
Angiolymphatic Invasion		
Y/N	8/101	7.3/92.3
Pleural invasion		
Y/N	33/76	30.3/69.7

The mean diameter of the tumours was 23.9 ± 15.6 mm. Sixteen patients (14.7%) had well differentiated ADC; 71 (65.1%) had moderately differentiated ADC; and 22 (20.2%) had poorly differentiated ADC (Table 2). STAS was observed in 70 cases (64.2%). The mean diameter of tumours showing STAS was significantly higher than that of the STAS-negative tumours (26.6 ± 15.3 vs 19.2 ± 15.03 mm; *P* = 0.01). In 41 cases (58.6%), STAS showed micropapillary features; in 15 (21.4%), it showed a solid pattern; and in 14 (20%), it occurred as single cells; 57 cases (81.4%) were classified as ‘low STAS’ and 13 (18.6%), as ‘high STAS’. Seventeen patients (15.6%) had lymph node involvement (N1 and N2); 13 of them (76.4%) had STAS. Lymphatic and pleural invasions were observed in 8 (7.3%) and 33 (30.3%) cases, respectively.

**Table 2:** Classification of histological subtypes

Histological subtypes	Number of patients	Percentage
Acinar	68	62.4
Lepidic	20	18.3
Papillary	6	5.5
Solid	8	7.4
Micropapillary	2	1.8
Cribriform	1	0.9
Mucin secreting	4	3.7

**Figure 2:**
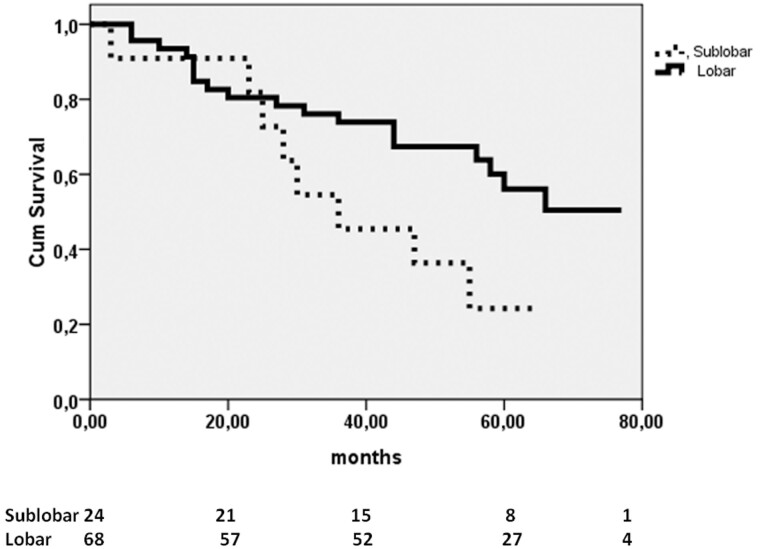
Kaplan–Meier curves of sublobar and major resections in patients N0 spread through air spaces-positive compared by the log-rank test (*P* = 0.04).

Overall 5-year and progression-free survival was 57 ± 5% and 53.6 ± 5%, respectively; the survival curves differed overall, with *P* = 0.042 and *P* = 0.027, respectively, with survival rates at 5 years higher in patients without STAS (72 ± 6% vs 46 ± 6% and 71 ± 5% vs 42.1 ± 4%). When we compared overall survival according to the type of resection (sublobar versus major resections), we found no significant difference between patients with and without STAS (*P* = 0.1). However, the presence of STAS in tumours smaller than 2 cm was a significant predictor of worse progression-free survival at 5 years in patients undergoing sublobar resection compared to those having major resections (52.6 ± 13.5% vs 89.7 ± 7%; *P* = 0.011). In addition, in N0 STAS-positive tumours, sublobar resection was associated with worse long-term survival compared to major resection (24.2 ± 13% vs 56.1 ± 8%; *P* = 0.04) (Fig. 2) and to a more negative trend regarding progression-free survival (60 ± 15% vs 79.9 ± 7%; *P* = 0.06). No significant difference in 5-year survival and progression-free survival was observed between patients with low STAS and those with high STAS.

With the χ^2^ test, age >70 years, the ground-glass opacity feature, diameter of the tumour > 20 mm, low grade of tumour differentiation, presence of STAS and a high STAS rate, adjuvant therapy, nodal involvement and recurrence were associated with a higher risk of death. Recurrence was associated with age >70 years, smoking pack-years >20, diameter of the tumour >20 mm, low grade of tumour differentiation, presence of STAS, nodal involvement and pleural invasion.

As reported in Table 3, Cox regression showed that age >70 years and recurrence were independent variables for survival, smoking pack-years >20, sublobar resection and nodal involvement were independent variables for recurrence and smoking pack-years >20, a history of cancer and pleural invasion were independent variables for local recurrence.

**Table 3:** Cox regression for survival, overall recurrences and local recurrences

Variables	*P*-value	HR	95% CI
Survival			
Age >70 years	0.04	0.867	0.031–1.318
Overall recurrence	<0.0001	2.647	1.143–5.762
Overall recurrences			
Pack-years >20	0.003	1.937	0.923–4.003
Sublobar resection	0.032	1.044	0.022–1.966
Nodal involvement	0.02	0.672	0.034–1.628
Local recurrences			
Pack-years >20	0.012	0.518	0.023–0.992
History of cancer	0.047	0.762	0.002–1.122
PLI	0.005	1.237	0.174–3.061

CI: confidence interval; PLI: pleural invasion; HR: Hazard Ratio.

## DISCUSSION

STAS is observed in a large percentage of patients with ADC [4]. Kadota *et al.* [1] in 2015 described STAS as a solid nest of tumour cells called a ‘tumour island’ in the alveolar spaces. Subsequently, several authors presented a number of retrospective studies identifying STAS as a negative prognostic factor impacting overall survival and onset of recurrence [5–13].

One of the goals of our study was to investigate whether STAS plays a role in determining the type of resection for patients with early-stage lung ADC. We have also investigated the impact of STAS on recurrence and survival in 2 different surgical settings: major lung resections and sublobar resections. We have stratified patients by TNM into 4 groups according to lymph node involvement (N0 or N1–N2) and STAS status (positive or negative), and we have compared them according to the type of surgical resection. We have assumed that in N0 patients, STAS could be an important prognostic factor for survival and recurrence. In fact, in locally advanced tumours, the presence of STAS does not affect prognosis in any group of patients (neither limited nor major resections); however, N0 patients with STAS undergoing sublobar resection showed an overall survival significantly worse and a higher risk of recurrence compared to N0 sublobar resections without STAS. According to our results, in patients with stage I lung ADC and STAS, a limited resection is associated with a significantly higher risk of recurrence and lower survival than a major resection. Kadota *et al.* [14] published similar conclusions in 2019; they found that in patients with STAS, limited resection was associated with a significantly higher risk of loco-regional recurrence than lobectomy.

Moreover, we have explored the synergistic effects of STAS and tumour size on progression free survival. Patients have been classified into 4 groups according to tumour size and STAS status. We found that in patients with small tumours (<20 mm), the presence of STAS strongly influenced (or significantly predicted) the onset of recurrence compared to tumours >20 mm. These data not only provide further evidence to support the impact of this pattern of local invasion but they also suggest that STAS could be considered as a parameter to be included in the staging system to predict prognosis, particularly in regard to smaller tumours. However, further studies on a large study sample are required to validate this hypothesis. An intriguing result of our study is the finding that STAS was more frequent than previously reported [2, 13]. We believe that this finding is related to our extensive sampling of the lung parenchyma surrounding the neoplastic lesion.

Dai *et al.* [6] reported that STAS could predict the prognosis of patients with ADC between 2 and 3 cm; furthermore, a larger study by Eguchi *et al.* [15] showed that the 5-year cumulative incidence of recurrence and the cumulative incidence of lung cancer-specific death in patients with an ADC up to 2 cm in diameter was stratified by STAS. Based on these data and on the published literature, we suggest that STAS should be included when assessing the prognosis of patients with ADC. Unfortunately, STAS cannot yet be identified reliably at the preoperative work-up, and the sensitivity and negative predictive value of frozen sections for STAS detection are still low [16].

### Limitations 

Some limitations of this study should be addressed, namely its single-centre retrospective nature and the relatively small population of patients; multicentre studies should be performed to confirm our results.

## CONCLUSIONS

This study provides preliminary evidence that STAS could be considered a new factor in a staging system to predict prognosis more accurately, particularly in patients with N0 and in tumours smaller than 2 cm. We have shown that in the subgroup of patients with ADC and STAS, limited resection is associated with a significantly higher risk of recurrence and lower survival; this observation should be considered when planning surgery.

## Funding

No funding was requested for this study.

**Conflict of interest:** none declared.

## Author contributions

**Sara Mantovani:** Data curation; Writing—original draft. **Angelina Pernazza:** Conceptualization; Data curation. **Massimiliano Bassi:** Formal analysis. **Davide Amore:** Data curation. **Jacopo Vannucci:** Validation; Writing—review & editing. **Camilla Poggi:** Investigation. **Daniele Diso:** Supervision. **Giulia d'Amati:** Data curation; Supervision; Validation; Writing—review & editing. **Carlo Della Rocca:** Supervision; Writing—review & editing. **Erino Angelo Rendina:** Supervision. **Federico Venuta:** Supervision. **Marco Anile:** Conceptualization; Data curation; Methodology; Validation; Writing—review & editing.

## Reviewer information

Interactive CardioVascular and Thoracic Surgery thanks Ayten Kayi Cangir, David G. Healy and the other, anonymous reviewer(s) for their contribution to the peer review process of this article.

## ABBREVIATIONS

ADC: Adenocarcinomas
STAS: Spread through air spaces
